# Mid-term functional and quality of life outcomes of robotic and laparoscopic ventral mesh rectopexy: multicenter comparative matched-pair analyses

**DOI:** 10.1007/s10151-021-02563-z

**Published:** 2021-12-21

**Authors:** K. E. Laitakari, J. K. Mäkelä-Kaikkonen, J. Kössi, M. Kairaluoma, S. Koivurova, L. Pollari, P. Ohtonen, T. T. Rautio

**Affiliations:** 1 Department of Surgery, Division of Gastroenterology, Oulu University Hospital, PO Box 21, 90029 OYS Oulu, Finland; 2 Medical Research Center Oulu, Center of Surgical Research, University of Oulu, Oulu, Finland; 3 Department of Surgery, Päijät-Häme Central Hospital, Lahti, Finland; 4 Department of Surgery, Keski-Suomi Central Hospital, Jyväskylä, Finland; 5 Department of Obstetrics and Gynecology, Oulu University Hospital, Oulu, Finland; 6 Division of Operative Care, Oulu University Hospital, Oulu, Finland; 7 The Research Unit of Surgery, Anesthesia and Intensive Care, University of Oulu, Oulu, Finland

**Keywords:** Robotic, Laparoscopic, Ventral rectopexy, Rectal prolapse, Incontinence, Obstructed defecation

## Abstract

**Background:**

The aim of this study was to compare patients’ mid-term functional and quality of life (QoL) outcomes following robotic ventral mesh rectopexy (RVMR) and laparoscopic ventral mesh rectopexy (LVMR).

**Methods:**

The data of consecutive female patients who underwent minimally invasive ventral mesh rectopexy for external or symptomatic internal rectal prolapse at 3 hospitals in Finland between January 2011 and December 2016 were retrospectively collected. Patients were matched by age and diagnosis at a 1:1 ratio. A disease-related symptom questionnaire was sent to all living patients at follow-up in July 2018.

**Results:**

After a total of 401 patients (RVMR, *n* = 187; LVMR, *n* = 214) were matched, 152 patients in each group were included in the final analyses. The median follow-up times were 3.3 (range 1.6–7.4) years and 3.0 (range 1.6–7.6) years for the RVMR and LVMR groups, respectively. The postoperative QoL measures did not differ between the groups. Compared with the LVMR group, the RVMR group had lower postoperative Wexner Incontinence Score (median 5 vs. median 8; *p* < 0.001), experienced significant ongoing incontinence symptoms less often (30.6% vs. 49.0%; *p* < 0.001) and reported less postoperative faecal incontinence discomfort evaluated with the visual analogue scale (median 11 vs. median 39; *p* = 0.005). RVMR patients had a shorter hospital stay (2.2 days vs. 3.8 days; *p* < 0.001) but experienced more frequent de novo pelvic pain (31.8% vs. 11.8%; *p* < 0.001).

**Conclusion:**

RVMR and LVMR patients had equal functional and QoL outcomes. Those who underwent RVMR had lower mid-term anal incontinence symptom scores but suffered more frequent de novo pelvic pain.

**Supplementary Information:**

The online version contains supplementary material available at 10.1007/s10151-021-02563-z.

## Introduction

The role of robotic surgery in treating external rectal prolapse (ERP) or symptomatic internal rectal prolapse (IRP) has not been demonstrated, and the impact on long-term outcomes remains unclear. Evidence of clinical outcomes is based mainly on non-randomised studies with relatively small study populations [1–4]. Only one randomised controlled trial (RCT) published to date has compared robotic ventral mesh rectopexy (RVMR) with laparoscopic ventral mesh rectopexy (LVMR) techniques for rectal prolapse in 30 patients [5]. A subsequent evaluation with the same study population revealed no differences in the quality of life (QoL) outcomes between the RVMR and LVMR procedures [6].

Comparative studies of the RVMR and LVMR procedures in terms of long-term functional outcomes are lacking. A prospective cohort study of 51 patients with ERP found no robust difference in short-term anal incontinence and obstructed defecation function between the two procedures [2]. In terms of perioperative and short-term outcomes, a recent review and meta-analysis of randomised and non-randomised studies comparing RVMR and LVMR for rectal prolapse reported that five studies with a total of 259 patients showed longer operating times and shorter hospital stays for RVMR procedures [7]. By contrast, no differences were noted in the conversion rates, morbidity or recurrence rates between RVMR and LVMR [7]. The prevailing data do not show a clear benefit for robotic surgery despite its acknowledged technical utility compared with conventional *straight stick* laparoscopy [8–10]. An important issue is whether the potential advantages result in better outcomes in terms of improved QoL and functional results.

The aim of this multi-center retrospective observational and questionnaire study was to compare the mid-term functional and QoL outcomes in a cohort of patients who underwent RVMR or LVMR operations for symptomatic internal or external rectal prolapse. Our secondary interest was to compare the perioperative and short-term postoperative courses in patients undergoing RVMR and LVMR.

## Materials and methods

### Study population and data collection

This is a comparative matched pairs study using registry and questionnaire data to evaluate mid-term functional and QoL outcomes after RVMR and LVMR. We retrospectively evaluated 401 consecutive female patients who underwent RVMR (*n* = 187) at Oulu University Hospital and LVMR (*n* = 214) at two central hospitals (Central Finland Central Hospital and Päijät-Häme Central Hospital) for ERP or symptomatic IRP between January 2011 and December 2016. The patients who underwent RVMR were matched by age (± 5 years) and diagnosis (ERP or IRP) at a 1:1 ratio to the patients who underwent LVMR; a total of 304 patients (152 in each group) were included in the final analyses. In two subpopulations, matching was performed in addition to age and diagnosis by indication of (1) incontinence and (2) obstructive defecation syndrome (ODS)/obstructed defecation. The indications for RVMR and LVMR and clinical follow-up were determined according to the individual centre’s practice. The only exclusion criterion was a redo rectopexy procedure. Data on demographic, perioperative and short-term outcomes were collected retrospectively from prospectively collected registry files of each institution and were collated into a single database. Additional data needed for analysis were retrieved from the patients’ medical records. The study protocol was approved by the Ethics Committees of Oulu University Hospital.

### Surgical technique

The robotic operations were performed at Oulu University Hospital, and the laparoscopic operations were performed at two central hospitals; all operations were conducted by experienced colorectal surgeons. The surgical technique in both methods followed the protocol described by D’Hoore and Penninckx [11], with slight modifications. All procedures were single mesh fixations to the posterior compartment. Suspension to the sacral promontory was performed with spiral attachments (Pro-Tack TM Fixation Device, Covidien), and the peritoneum was closed over the mesh with a continuous suture. The robotic operations were performed with a Da Vinci Si surgical system (Intuitive Surgical Inc., Sunnyvale, CA, USA), and the same protocols were followed.

### Outcomes

We collected the following data on patient-related characteristics: age, body mass index (BMI), the American Society of Anesthesiologists (ASA) classification, indications/symptoms for operation, anatomic diagnosis, previous hysterectomy and previous pelvic surgery.

The intra- and postoperative parameters compared between RVMR and LVMR were as follows: operating time, operating theatre time, intraoperative bleeding, conversion to an open technique, mortality, length of hospital stay, complications, recurrence rate and reoperations.

Mid-term outcomes were assessed retrospectively using a questionnaire sent to all women still living at the follow-up date in July 2018 (Supplementary material, Questionnaire). After this, matching by age and diagnosis was performed, and the results between RVMR and LVMR were compared. The follow-up questionnaire included the Wexner Continence Grading Scale [12] for incontinence symptoms (range: 0–20), with a score > 9 regarded as disturbing incontinence. The ODS score [13] was used for constipation/obstructed defecation symptoms (range: 0–40), with a score > 20 regarded as disturbing constipation. The possible discomfort experienced because of incontinence or obstructed defecation symptoms was evaluated with a 100 mm visual analogue scale (VAS; 0–100, no discomfort–great discomfort). The changes in/defecatory symptoms before and after the operation were also evaluated using a VAS scale (0–100, much worse–much better), and patients marking a point at 61–100 mm (worse–better) on the VAS scale were considered to have experienced an improvement in defecatory symptoms. The effect of postoperative symptoms on QoL was queried (VAS; 0–100, much worse–much better), and patients marking a point at 61–100 mm (worse–better) on the VAS scale were considered to have experienced an improved QoL. Questions on the appearance of de novo symptoms during the first 6 months postoperative included urinary incontinence, incomplete bladder emptying and pelvic pain, and the patients were also free to comment on additional symptoms. The impact of the operation on sexual life (VAS; 0–100, much worse–much better) was also queried. The patients were asked whether they were satisfied with the operation result (yes/no/cannot say).

### Statistical analysis

Summary statistics are presented as mean with standard deviation (SD) or as median with 25th–75th percentiles, unless otherwise stated. Matched pair analyses were performed using a linear mixed model for continuous variables and a generalised linear mixed model for categorical variables. In both models, matched pairs were used as random effects.

Two-tailed *p *values were presented, and all statistical analyses were performed using SAS (version 9.4, SAS Institute Inc., Cary, NC, USA) or SPSS for Windows (IBM Corp., Released 2017. IBM SPSS Statistics for Windows, Version 25.0. IBM Corp, Armonk, NY, USA.).

## Results

### Patient demographics

Originally, the data of 401 patients (RVMR, *n* = 187; LVMR, *n *= 214) were collected. After age and diagnosis matching, a total of 152 patients for both RVMR and LVMR groups were included in the analyses. Of these 152 pairs, 36 had ERP and 116 had IRP diagnoses. The matched groups did not differ in terms of BMI, ASA, previous hysterectomy or previous pelvic surgery. Obstructive defecation symptoms either alone or combined with rectal prolapse were equally common in both groups. Although the patients in the RVMR group were statistically significantly younger (mean age: 62.7, SD: 13.7 vs. mean age: 63.9 years, SD 14.2; *p* < 0.001), the difference was not clinically significant. The patients in the LVMR group presented more frequently with rectal prolapse without functional symptoms (LVMR: 23.0% vs. RVMR: 8.6%, *p* < 0.001) and with isolated anal incontinence (LVMR: 34.9% vs. RVMR: 7.2%, *p* < 0.001) than did the patients in the RVMR group. The number of patients having obstructed defecation symptoms combined with anal incontinence was higher in the RVMR group (RVMR: 18.4% vs. LVMR: 2.6%, *p* < 0.001). The baseline characteristics are presented in Table 1. The response rates on the follow-up questionnaires sent in July 2018 were 75.8% (138/182 living patients) and 68.2% (133/195 living patients) for the RVMR and LVMR groups without matching, respectively. The median follow-up time from the operation to the follow-up questionnaires sent in July 2018 was 3.3 (range, 1.6–7.4) years for the RVMR group and 3.0 (range: 1.6–7.6) years for the LVMR group (*p* = 0.34). Respondents and non-respondent women did not differ statistically in terms of age, BMI, ASA, preoperative ODS or preoperative Wexner (Supplementary material, Table 6).

**Table 1:** Baseline characteristics

	RVMR No. 152	LVMR No. 152	*p*
Age (years)	62.7 ± 13.7	63.9 ± 14.2	< 0.001
Body mass index	26.4 ± 4.4	26.3 ± 4.8	0.81
ASA class			> 0.9
1	33 (21.7)	58 (38.7)	
2	78 (51.3)	51 (34.0)	
3	37 (24.3)	34 (22.7)	
4	4 (2.6)	7 (4.7)	
Indication/symptom			
Prolapse only	13 (8.6)	35 (23.0)	< 0.001
Prolapse and ODS	4 (2.6)	1 (0.7)	0.43
Prolapse and incontinence	12 (7.9)	0 (0)	n.d
ODS only	72 (47.4)	58 (38.2)	0.10
Incontinence only	11 (7.2)	53 (34.9)	< 0.001
ODS and incontinence	28 (18.4)	4 (2.6)	< 0.001
Previous hysterectomy	56 (36.8)	65 (44.2)	0.19
Previous pelvic surgery	38 (25.0)	52 (34.2)	0.08

Nominal variables are reported as counts and percentages (in parentheses); continuous variables are reported as mean and standard deviation

*ASA* American Society of Anesthesiologists; *ODS* obstructed defecation syndrome; *RVMR* robotic ventral mesh rectopexy; *LVMR* laparoscopic ventral mesh rectopexy; *ERP* external rectal prolapse; *IRP* internal rectal prolapse; *n.d.* not definable

### Mid-term functional and quality of life outcomes

The patient-reported mid-term functional outcomes are presented in Table 2. Patients reported lower Wexner scores for faecal incontinence after RVMR than after LVMR (median: 5 vs. median: 8, *p* < 0.001), and fewer patients experienced significant ongoing incontinence symptoms after RVMR (30.6% vs. 49.0%, *p* < 0.001). The discomfort of faecal incontinence (measured with VAS; 0–100) was also lower after RVMR than after LVMR (median: 11 vs. median: 39, *p* = 0.005). No differences were detected for the ODS score, ongoing obstructed defecation symptoms or obstructed defecation discomfort (measured with VAS; 0–100) measures between the groups. The defecatory symptom change and the improvement in postoperative defecatory symptoms were similar for both groups. The postoperative QoL, the experience of QoL improvement and postoperative contentment with sexual life were also comparable between the groups. No differences were found for the de novo symptoms in terms of urinary incontinence or urinary retention between the groups. However, de novo pelvic pain occurred more often after RVMR than after LVMR. Amongst all RVMR respondents, 58.9% were subjectively satisfied with the operation, 15.2% were dissatisfied and 25.9% could not say. Amongst LVMR patients, 57.8% were satisfied, 14.7% were dissatisfied and 27.5% could not say. The differences between the study groups in terms of satisfaction rates were not statistically significant (*p* = 0.87).

**Table 2:** Mid-term functional outcome comparison

	RVMR	LVMR	*p*	Difference^1^/OR^2^	95% CI
Wexner score^a,b^, median (range)	5 (2–10)	8 (4–14)	< 0.001	− 2.5^1^	− 3.9 to − 1.0
Significant ongoing incontinence symptoms (Wexner > 9)^c^, *n*(%))	34 (30.6)	51 (49.0)	< 0.001	0.46^2^	0.3 to 0.8
Faecal incontinence discomfort (VAS)^b,d^, median (range)	11 (1–49)	39 (6–69)	0.005	− 12.6^1^	− 21.3 to − 3.9
ODS score^b,e^, median (range)	13 (8–20)	12 (7–17)	0.11	1.7^1^	− 0.4 to 3.7
Significant ongoing OD symptoms (ODS > 20)^c^, n(%)	25 (22.1)	14 (13.5)	0.35	1.8^2^	0.89 to 3.8
Obstructed defecation discomfort (VAS)^b, d^, median (range)	56 (18–86)	43 (18–71)	0.13	6.9^1^	− 2.1 to 16.0
Defecatory symptom change (VAS)^b, f^, median (range)	72 (50–88)	72 (52–89)	> 0.9	− 0.29^1^	− 7.7 to 7.1
Defecatory symptom improvement (VAS 61–100)^c^, *n*(%)	67 (62.6)	68 (67.3)	0.61	0.81^2^	0.46 to 1.4
Postoperative QoL (VAS)^b,f^, median (range)	72 (50–89)	77 (54–89)	0.74	− 1.2^1^	− 8.4 to 6.0
Experience d QoL improvement (VAS 61–100) ^c, f^, *n*(%)	70 (64.8)	65 (63.7)	0.87	1.0^2^	0.59 to 1.9
Postoperative contentment with sexual life (VAS)^b, f^, median (range)	54 (47–78)	55 (46–82)	0.89	0.71^1^	− 9.5 to 10.9
De novo urinary incontinence^c^, *n*(%)	21 (19.1)	23 (22.6)	0.53	0.81^2^	0.4 to 1.6
De novo urinary retention^c^, *n*/(%)	21 (19.1)	15 (14.7)	0.55	1.4^2^	0.66 to 2.8
De novo pelvic pain^c^, *n*(%)	35 (31.8)	12 (11.8)	< 0.001	3.5^2^	1.7 to 7.3
Satisfaction with operation, *n*(%)			0.87	1.0^3^	0.60 to 1.8
No^c^	17 (15.2)	15 (14.7)			
Yes^c^	66 (58.9)	59 (57.8)			
Cannot say^c^	29 (25.9)	28 (27.5)			
Follow-up period; years, median, SD	3.3 (1.4)	3.0 (2.1)	0.34	− 0.2^1^	− 0.6 to 0.2
Range	1.6–7.4	1.6–7.6			

After matching RVMR patients by age and diagnosis to the LVMR patients, 152 pairs were detected

*RVMR* robotic ventral mesh rectopexy; *LVMR* laparoscopic ventral mesh rectopexy; *ERP* external rectal prolapse; *IRP* internal rectal prolapse; *CI* confidence interval; *QoL* quality of life

^a^Wexner score for faecal incontinence (minimum–maximum; 0–20)

^b^Values are reported as medians with 25th and 75th percentiles

^c^Values are reported as counts and percentiles

^d^VAS: visual analogue scale (no discomfort–great discomfort; 0–100)

^e^ODS: obstructed defecation symptom score (minimum–maximum; 0–40)

^f^ VAS: visual analogue scale (much worse–much better; 0–100)

^1^Difference between means

^2^OR: odds ratio (reference group: LVMR)

^3^OR: odds ratio (reference group: LVMR) for not satisfied with operation/cannot say

Table 3 presents the mid-term functional outcome comparison of incontinence and obstructed defecation results in two subpopulations. After further matching by age, diagnosis, and indication, 119 pairs with incontinence and 134 pairs with ODS were detected. Wexner scores for faecal incontinence (median 5 vs. median 7, *p* = 0.032) were lower after RVMR than after LVMR. No differences were found in the ODS score, significant ongoing symptoms, discomfort of faecal incontinence or obstructed defecation, defecatory symptom change, or defecatory symptom improvement measures between the groups.

**Table 3:** Mid-term functional outcome comparison, in which incontinence and obstructed defecation results were matched by age, diagnosis and indication

	RVMR	LVMR	*p*	Difference^1^/ OR^2^	95% CI
Wexner score^a,b,^, median (range)	5 (2–12)	7 (4–13)	0.032	− 1.7^1^	− 3.3 to − 0.1
Significant ongoing incontinence symptoms (Wexner > 9)^c^, *n*(%)	27 (32.9)	33 (44.0)	0.16	0.6^2^	0.3 to 1.2
Faecal incontinence discomfort (VAS)^b,d^, median (range)	14 (2–56)	32 (3–63)	0.19	− 6.7^1^	− 16.8 to 3.4
ODS score^b,e^	12 (8–20)	12 (7–17)	0.20	1.4^1^	− 0.7 to 3.5
Significant ongoing OD symptoms (ODS > 20)^c^, *n*(%)	24 (23.3)	14 (15.9)	0.21	1.6^2^	0.8 to 3.4
Obstructed defecation discomfort (VAS)^b, d^, median (range)	55 (15–85)	43 (18–71)	0.16	6.9^1^	− 2.8 to 16.6
Defecatory symptom change (VAS)^b, f^, median (range)	72 (50–88)	70 (51–87)	0.76	1.2^1^	− 6.8 to 9.2
Defecatory symptom improvement (VAS 61–100)^c^, *n*(%)	59 (60.8)	55 (64.7)	0.59	0.85^2^	0.46 to 1.6

Wexner/incontinence results were matched by age, ERP/IRP and indication (incontinence) (*n* = 119 pairs). ODS/obstructed defecation results were matched by age, ERP/IRP and indication (ODS) (*n* = 134 pairs)

*RVMR* robotic ventral mesh rectopexy; *LVMR* laparoscopic ventral mesh rectopexy; *ERP* external rectal prolapse; *IRP* internal rectal prolapse; *CI* confidence interval; *QoL* quality of life

^a^Wexner score for faecal incontinence (minimum–maximum; 0–20)

^b^Values are reported as medians with 25th and 75th percentiles

^c^Values are reported as counts and percentiles

^d^VAS: visual analogue scale (no discomfort–great discomfort; 0–100)

^e^ODS: obstructed defecation symptom score (minimum–maximum; 0–40)

^f^ VAS: visual analogue scale (much worse–much better; 0–100)

^1^Difference between means

^2^OR: odds ratio (reference group: LVMR)

^3^OR: odds ratio (reference group: LVMR) for not satisfied with operation

### Recurrence and reoperations

Data on the recurrence of prolapse were available for 50% of the patients with ERP. Recurrent prolapse was observed in 3/17 (17.6%) and 2/19 (10.5%) patients in the RVMR and LVMR groups (*p* = 0.55), respectively. The recurrence rate of IRP was not analysed. Reoperation data are presented in Table 4. During the study period, 9 (5.9%) reoperations were performed in the RVMR group and 13 (9.0%) in the LVMR group, with no difference noted between the study groups (*p* = 0.31). One patient (0.7%) in the RVMR group (with IRP) and two patients (1.4%) in the LVMR group underwent reoperations during the follow-up period with a redo ventral rectopexy technique.

**Table 4:** Reoperations

Reoperation after surgery	RVMR No. 152	LVMR No. 152	*p*	OR	95% CI
Reoperation total	9 (5.9)	13 (9.0)	0.31	0.63	0.26 to 1.5
D’Hoore LVMR	1 (0.7)	2 (1.4)			
Delorme	1 (0.7)	5 (3.5)			
Others	5 (3.3)	5 (3.5)			
Posterior colporrhaphy	2 (1.3)	1 (0.7)			

Variables are reported as counts and percentages (in parentheses)

*RVMR* robotic ventral mesh rectopexy; *LVMR* laparoscopic ventral mesh rectopexy

### Perioperative details and short-term outcomes

The perioperative data and short-term postoperative outcomes are summarised in Table 5. The operating time (mean: 116 min vs. mean: 135 min, *p* < 0.001) and in-hospital stay (mean: 2.2 days vs. mean: 3.8 days, *p* < 0.001) were shorter in the RVMR group. No conversions were needed in the robotic operations, whereas two laparoscopic operations (2.7%) were converted to open surgery. A total of 32 (21.1%) patients faced postoperative complications after RVMR and 12 patients (7.9%) after LVMR (*p* = 0.002), but serious surgical complications (Clavien–Dindo grade ≥ 3) were at the same level (RVMR: 2.6% vs. LVMR: 4.6%, *p* = 0.36). Only one mesh-related complication occurred in the whole study; this was mesh erosion through the vaginal wall in one member of the RVMR group. The small protruding part of the mesh was resected transvaginally. No postoperative mortality occurred in either group.

**Table 5:** Perioperative data and short-term postoperative outcomes

	RVMR No. 152	LVMR No. 152	*p*	Difference/OR^1^	95% CI
Operating time (min)	116 ± 30	135 ± 47	< 0.001	− 18.3	− 24.1 to − 9.4
Operating theatre time (min)	194 ± 36	183 ± 33	0.06	10.5	− 0.6 to 21.7
Bleeding (ml)	19 ± 64	32 ± 56	0.09	− 13.4	− 28.7 to 1.9
Conversion	0	2 (2.7)	n.d	n.d	n.d
Mortality	0	0	n.d	n.d	n.d
In-hospital stay, (days)	2.2 ± 1.2	3.8 ± 2.2	< 0.001	− 1.7	− 2.1 to − 1.3
Complications	32 (21.1)	12 (7.9)	0.002	3.1^1^	1.5 to 6.3
Pneumonia	0	1 (0.5)			
Pulmonary embolism	0	1 (0.5)			
Urinary tract infection	1 (0.5)	3 (1.4)			
Urinary retention	13 (7.0)	0			
Chronic pelvic pain	1 (0.5)	1 (0.5)			
Ileus	1 (0.5)	0			
Bleeding	2 (1.1)	4 (1.9)			
Rectal perforation	0	1 (0.5)			
Vaginal perforation	1 (0.5)	3 (1.4)			
Bowel perforation	1 (0.5)	3 (1.4)			
Mesh-related complication	1 (0.5)	0			
Ureter lesion	0	1 (0.5)			
Abdominal pain	4 (2.1)	0			
Wound haematoma	2 (1.1)	0			
Postoperative fewer	0	1 (0.5)			
Incisional hernia	0	1 (0.5)			
Others	8 (4.3)	0			
Clavien–Dindo ≥ 3 complications	4 (2.6)	7 (4.6)	0.36	0.56^a^	0.16 to 2.0

Nominal variables are reported as counts and percentages (in parentheses); continuous variables are reported as mean and standard deviation

^a^*OR* odds ratio; *RVMR* robotic ventral mesh rectopexy; *LVMR* laparoscopic ventral mesh rectopexy; *CI* confidence interval; n.d.: not definable

## Discussion

To the best of our knowledge, this series of 304 patients is the largest comparative outcome study with the longest follow-up time reported to date. Probably the most important finding of our study was that RVMR and LVMR gave equal outcomes on the mid-term overall QoL. An interesting finding was that the patients had better symptom scores in anal incontinence, but they experienced more frequent de novo pelvic pain after RVMR than after LVMR. In terms of perioperative and short-term outcomes, RVMR showed the benefits of a shorter length of hospital stay and shorter operating time. However, RVMR was surprisingly associated with more minor complications compared with LVMR.

Our primary hypothesis was that the potential technical advantages of robotic surgery when operating in a deep and narrow pelvis could lead to a more precise dissection and mesh attachment and thereby a better long-term outcome. However, this hypothesis proved to be only partly true. According to our study, the patients who underwent RVMR had better mid-term anal continence outcomes than those who underwent LVMR, but the two groups had obstructed defecation symptoms and new-onset symptoms. Our findings differ slightly from those reported previously in the literature. Only two previous studies have compared the long-term functional outcome after RVMR and LVMR for rectal prolapse operations conducted, as described by D’Hoore and Penninckx [11]. A small cohort study conducted by Mantoo et al. [1], with 44 RVMR and 74 LVMR performed for various indications (follow-up time of 16 ± 7 months), reported comparable improvements in the postoperative Wexner scores between the RVMR and LVMR groups, as well as a greater reduction in ODS scores after RVMR. Another prospective short-term cohort study with 17 RVMR and 34 LVMR procedures (follow-up time of 12 months) performed for ERP found an equal long-term postoperative faecal incontinence severity score between RVMR and LVMR [2]. The true difference in anal incontinence outcome between the RVMR and LVMR groups may be confounded, as both the postoperative and preoperative median Wexner incontinence scores were lower in the RVMR group [2]. The reason for the discrepancy between our findings and those of previous studies is not evident. However, the longer follow-up time, different patient populations and variations in the study settings may have contributed to the different study outcomes.

We did not find any difference between the study groups in terms of the QoL evaluated with the VAS scale in the postoperative symptom questionnaire. Previous data comparing long-term QoL results after RVMR and LVMR for rectal prolapse are scarce. In a medium-term study by Mehmood et al. [2], a postoperative QoL questionnaire (SF-36) gave a slightly higher median physical component score favouring RVMR at 12 months postoperatively. Our recent study, which included 30 patients (24 IRP and 6 ERP cases), detected no difference between RVMR and LVMR in terms of QoL 24 months after operation, as measured with the 15D instrument [6]. The current study presents the QoL results with the longest follow-up time so far, bearing in mind that the method to study the issue was crude. The reason for the equal benefit in QoL between the groups could be the outcome being balanced by different effects on anal continence and ODS symptoms.

In our study, the recurrence rate of ERP was 17.6% after RVMR and 10.5% after LVMR, with no statistically significant difference between the study groups. The reoperation rates between RVMR and LVMR were also comparable. These results are in line with the prevailing literature comparing robotic and laparoscopic techniques, as similar recurrence rates were reported between RVMR (0–7%) and LVMR (0–8%) [7, 14]. A meta-analysis of four studies involving 104 RVMR and 168 LVMR patients detected no difference in reoperations between the two procedures [15].

In earlier studies, RVMR and LVMR were shown to have either similar postoperative complication rates [2–5], or RVMR had a more favourable outcome [1, 15]. We unexpectedly found an increased risk of postoperative complications after RVMR, but the procedures had equally low rates of Clavien–Dindo ≥ 3 complications. The reason for the more frequent complication rate in the RVMR group is not clear, but it may reflect differences in the accuracy of recording postoperative data in the different hospitals.

Previous studies have reported comparable rates of conversion for RVMR and LVMR, ranging from 0% to 2% for RVMR and 0% to 4% for LVMR [1–5]. We had similar results in our study, as the conversion rates were 2.7% (2/152) for LVMR and 0% for RVMR. The number of conversion incidences was relatively small in both groups.

In our study, the length of hospital stay was shorter for the RVMR group (− 1.7 days) than for the LVMR group. The participating hospitals all used the Enhanced Recovery After Surgery protocol; thus, no reasons related to the operational model used would explain the differences in hospital stays. Similarly, two previous meta-analyses, with 221 [15] and 259 [7] patients, found statistically significant reductions in the length of stay in RVMR operations but only by 0.33 [15] and 0.36 [7] days.

In contrast to our finding of a shorter RVMR operating time (− 18.3 min), a previous meta-analysis by Albayati et al. [7] showed a statistically significant increase in RVMR operating time, with a mean weighted difference of 22.88 min.

Several limitations should be noted when interpreting the results of our study. This study was limited by its retrospective and cross-sectional nature with no in-person follow-up visits and inadequate ERP recurrence follow-up data. Even if both the ODS and the Wexner constipation scores in Finnish have been reviewed by a language translation agency, one of the limitations of our paper is that the Wexner and ODS questionnaires have not been validated in Finnish. The absence of validated questionnaires (Wexner and ODS scores) before the surgery does not allow to compare the symptoms before and after surgery in the two groups. The loss of patients to follow-up was notable and led to a suboptimal questionnaire response rate. No systematic follow-up was conducted for patients without symptoms. Patients were asked about the de novo pelvic symptoms using separate questionnaire without definite specification or duration; therefore, strong conclusions about the differences between the groups cannot be drawn. In addition, the operations were performed by different surgeons and different times. The robotic operations were performed in a single tertiary referral centre. The strengths of this study were its relatively long follow-up time and the high number of patients, even exceeding the number of patients included in the meta-analyses published thus far.

## Conclusion

RVMR and LVMR gave equal outcomes for QoL. The patients had lower mid-term anal incontinence symptom scores after RVMR than after LVMR. The findings of this study indicate that RVMR may offer better postoperative recovery, but its true possible benefit in terms of long-term outcomes remains unclear.

## Supplementary Information

Below is the link to the electronic supplementary material.

Supplementary file1 (DOCX 15 KB)

Supplementary file2 (DOCX 77 KB)
