# Supplementary material for: Mid-term functional and quality of life outcomes of robotic and laparoscopic ventral mesh rectopexy: multicenter comparative matched-pair analyses
Source: Tech Coloproctol. 2021 Dec 21;26(4):253–60. doi: 10.1007/s10151-021-02563-z (PMC8917003; doi:10.1007/s10151-021-02563-z)
Supplement: Supplementary file 1 — Supplementary file1 (DOCX 15 KB) [file 10151_2021_2563_MOESM1_ESM.docx]

**Table 6.** Baseline characteristics on respondents and non-respondents.

|  | *Respondents*  *No. 271* | *Non-respondents*  *No. 106* |
| --- | --- | --- |
| Age (years) | 62.1 (13.1) | 63.1 (17.5) |
| Body mass index | 26.4 (4.6) | 26.0 (4.2) |
| ASA class |  |  |
| 1 | 82 (30.4) | 29 (27.6) |
| 2 | 129 (47.8) | 52 (49.5) |
| 3 | 54 (20.0) | 20 (19.0) |
| 4 | 5 (1.9) | 4 (3.8) |
| Preoperative ODS | 21.2 (7.2) | 22.2 (8.1) |
| Preoperative Wexner | 11.3 (5.7) | 9.9 (6.7) |
|  |  |  |

Nominal variables are reported as counts and percentages (in parentheses); continuous variables are reported as mean and standard deviation; ASA: American Society of Anesthesiologists; ODS: obstructed defecation syndrome; Wexner: Wexner score for fecal incontinence.
