# Supplementary material for: Mid-term functional and quality of life outcomes of robotic and laparoscopic ventral mesh rectopexy: multicenter comparative matched-pair analyses
Source: Tech Coloproctol. 2021 Dec 21;26(4):253–60. doi: 10.1007/s10151-021-02563-z (PMC8917003; doi:10.1007/s10151-021-02563-z)
Supplement: Supplementary file 2 — Supplementary file2 (DOCX 77 KB) [file 10151_2021_2563_MOESM2_ESM.docx]

Postoperative symptom questionnaire

Study code no.

Date______________________

When answering each question, please choose the option that best describes your situation. It is very important that you answer every question.

1. Answer the following questions by circling the alternative that best describes you for each question.

| Question/points | 0 | 1 | 2 | 3 | 4 |
| --- | --- | --- | --- | --- | --- |
| Incontinence to solid stool | Never | Less than once a month | Less than once a week but at least once a month | Less than once a day but at least once a week | At least once a day |
| Incontinence to liquid stool | Never | Less than once a month | Less than once a week but at least once a month | Less than once a day but at least once a week | At least once a day |
| Incontinence to gas | Never | Less than once a month | Less than once a week but at least once a month | Less than once a day but at least once a week | At least once a day |
| Wears pads | Never | Less than once a month | Less than once a week but at least once a month | Less than once a day but at least once a week | At least once a day |
| Lifestyle alteration | Never | Less than once a month | Less than once a week but at least once a month | Less than once a day but at least once a week eek | At least once a day |

2. How much inconvenience do you suffer from stool incontinence? Mark a cross (X) on the line describing the amount of inconvenience you have experienced.

No inconvenience at all ________________________________­­_______ Very much inconvenience

3. Do you have constipation or difficulty defecating?

Yes No

Version 20/ 12 / 2017

4. Answer the questions in the table by placing a cross (X) in the box that best describes your bowel function over the past 6 months.

|  | Yes | No |
| --- | --- | --- |
| I defecate less than three times a week |  |  |
| My stools are rarely loose without laxatives (stool softeners) |  |  |
| Lumpy or hard stools in at least 25% of defecations |  |  |
| I don't feel the need to defecate |  |  |
| Straining during at least 25% of defecations |  |  |
| Sensation of anorectal obstruction/blockage for at least 25% of defecations |  |  |
| Sensation of incomplete evacuation for at least 25% of defecations |  |  |
| Manual manoeuvres to facilitate at least 25% of defecations (e.g. digital evacuation, support of the pelvic floor) |  |  |

5. Answer the following questions about difficulty defecating by placing a cross (X) in the box that best describes you (ODS score).

| a. Defecation frequency | | | | | | | |  | | |  | |  |  | | | | | |  | | |  |
| --- | --- | --- | --- | --- | --- | --- | --- | --- | --- | --- | --- | --- | --- | --- | --- | --- | --- | --- | --- | --- | --- | --- | --- |
| 0 | |  | |  | 1–2 defecations per 1–2 days | | |  | | |  | |  |  | | | | | |  | | |  |
| 1 | |  | |  | 2 defecations per week or 3 defecations/attempts per day | | | | | | | | | | | | |  | |  | | |  |
| 2 | |  | |  | 1 defecation per week or 4 defecations/attempts to defecate per day | | | | | | | |  |  | | | | | |  | | |  |
| 3 | |  | |  | Less than 1 defecation per week or more than 4 defecations/attempts per day | | | | | | | | | | | | | | | | | |  |
|  | |  | |  |  | |  | |  |  | |  | | |  |  | | | | | |  | |
| b. Straining intensity | | | | | | | | | | | | | | | | | |  | |  | | |  |
| 0 | |  | |  | | None or light | | |  |  | |  | | |  |  | | | | | |  | |
| 1 | |  | |  | | Moderate | | |  |  | |  | | |  |  | | | | | |  | |
| 2 | |  | |  | | Intensive | | |  |  | |  | | |  |  | | | | | |  | |
| c. Straining extension | | | | | | | | | | |  | |  |  | | | | | |  | | |  |
| 0 | |  | |  | | No straining | | |  |  | |  | | |  |  | | | | | |  | |
| 1 | |  | |  | | Short time |  | |  |  | |  | | |  |  | | | | | |  | |
| 2 | |  | |  | | Prolonged | | | | | | |  |  | | | | | |  | | |  |
|  | |  | |  | |  |  | |  |  | |  | | |  |  | | | | | |  | |
| d. Sensation of incomplete evacuation | | | | | | | | | | | | | | | | | | | | | | |  |
| 0 | |  | |  | | Never | | |  |  | |  | | |  |  | | | | | |  | |
| 1 | |  | |  | | Once a week or less | |  | | |  | |  |  | | | | | |  | | |  |
| 2 | |  | |  | | Twice a week | | |  |  | |  | | |  |  | | | | | |  | |
| 3 | |  | |  | | More than twice a week | | | | |  | |  |  | | | | | |  | | |  |
|  | |  | |  | |  | |  |  |  | |  | | |  |  | | | | | |  | |
| e. Rectal/perineal pain or discomfort? | | | | | | | | | | | | |  |  | | | | | |  | | |  |
| 0 | |  | |  | | Never | | |  |  | |  | | |  |  | | | | | |  | |
| 1 | |  | |  | | Once a week or less | |  | | |  | |  |  | | | | | |  | | |  |
| 2 | |  | |  | | Twice a week | | |  |  | |  | | |  |  | | | | | |  | |
| 3 | |  | |  | | More than twice a week | |  |  |  | |  | | |  |  | | | | | |  | |
|  | |  | |  | |  | |  |  |  | |  | | |  |  | | | | | |  | |
| f. Activity reduction per week | | | | | | | | |  |  |  | | | | | |  | |  |  | | |  |
| 0 | |  | |  | | Never | |  |  |  | |  | | |  |  | | | | | |  | |
| 2 | |  | |  | | < 25% of activity | |  |  |  | |  | | |  |  | | | | | |  | |
| 4 | |  | |  | | 25-50% of activity | |  |  |  | |  | | |  |  | | | | | |  | |
| 6 | |  | |  | | > 50% of activity | |  |  |  | |  | | |  |  | | | | | |  | |
|  | |  | |  | |  | |  |  |  | |  | | |  |  | | | | | |  | |
| g. Laxatives | | | | | | | | | | | | | | | | | | | | | | |  |
| 0 | |  | |  | | Never | | |  |  | |  | | |  |  | | | | | |  | |
| 1 | |  | |  | | < 25% of defecations | |  | | |  | |  |  | | | | | |  | | |  |
| 3 | |  | |  | | 25–50% of defecations | |  | | |  | |  |  | | | | | |  | | |  |
| 5 | |  | |  | | > 50% of defecations | |  | | |  | |  |  | | | | | |  | | |  |
| 7 | |  | |  | | Always | |  |  |  | |  | | |  |  | | | | | |  | |
|  | |  | |  | |  | |  |  |  | |  | | |  |  | | | | | |  | |
| h. Enemas | | | | | | | | | | | | |  |  | | | | | |  | | |  |
| 0 | |  | |  | | Never | | |  |  | |  | | |  |  | | | | | |  | |
| 1 | |  | |  | | < 25% of defecations | |  | | |  | |  |  | | | | | |  | | |  |
| 3 | |  | |  | | 25–50% of defecations | |  | | |  | |  |  | | | | | |  | | |  |
| 5 | |  | |  | | > 50% of defecations | | | | |  | |  |  | | | | | |  | | |  |
| 7 | |  | |  | | Always | |  |  |  | |  | | |  |  | | | | | |  | |
|  | |  | |  | |  | |  |  |  | |  | | |  |  | | | | | |  | |
| i. Digitation | | | | | | | | | | | | | | | | | |  | |  | | |  |
|  |  | |  | | |  | |  |  |  | |  | | |  | | | | | |  |  | |
| 0 |  | |  | | | Never | | |  |  | |  | | |  | | | | | |  |  | |
| 1 |  | |  | | | < 25% of defecations | |  | | |  | |  | | | | |  | |  | | |  |
| 3 |  | |  | | | 25–50% of defecations | |  | | |  | |  | | | | |  | |  | | |  |
| 5 |  | |  | | | > 50% of defecations | |  | | |  | |  | | | | |  | |  | | |  |
| 7 |  | |  | | | Always | |  |  |  | |  | | |  | | | | | |  |  | |

1. How much do you suffer from constipation/defecation difficulties? Put a cross (X) on the line to describe the inconvenience you have experienced.

No inconvenience at all ________________________________________ Very much inconvenience

1. Compare the fecal symptoms that occurred before surgery with the current situation after surgery. Put a cross (X) on the line that describes your current symptoms situation.

Much worse _________________________________________ Much better

1. Evaluate the impact of symptoms on your quality of life. What has your quality of life been like after surgery? Put a cross (X) on the line that describes the quality of life you are experiencing.

Much worse _________________________________________ Much better

1. Do you have an active sexual life?

No (please move to the next question)

Yes If yes, evaluate the impact of the surgery on your sexual life:

After the operation, my sexual life has been: put a cross (X) on the line that describes

your satisfaction with your sexual life after surgery.

Much worse ________________________________________ Much better

1. During the 6 months after the operation, did you develop any new pelvic symptoms that did NOT occur before the operation?

No (please move to the next question)

Yes Choose from below:

1. urinary incontinence when straining

2. problems with bladder emptying

3. pelvic pain

4. no sense of defecation need

5. hurry to the toilet when the need to defecate arises

something else? ____________________________________________________________________

1. Did you have any other pelvic floor symptoms before surgery? If yes, put a cross (X) on the line that describes the change in your symptom after rectopexy.

a. Urinary incontinence Yes No symptom

Much worse ________________________________________ Much better

b. Problems with bladder emptying Yes No symptom

Much worse _______________________________________ Much better

c. Pelvic floor or rectal pain Yes No symptom

Much worse ________________________________­­­_______ Much better

d. A feeling of bulge because of gynecological prolapse (women)

Yes No symptom

Much worse ________________________________________ Much better

e) other effect? ________________________________________________________

12. Have you had any pelvic pain in the last week?

No pain at all _________________________________________ Very severe pain

13.Are you satisfied with the result of the rectopexy?

Yes No Cannot say

14. If you could choose again now, would you still choose rectopexy?

Yes No

Thank you for your answers. They will be handled with the utmost confidentiality.

Please return the completed questionnaire and the signed consent form in the enclosed envelope by­­­­_____________________.
